# “Longitudinal Fecal Microbiome Study of Total Body Irradiated Mice Treated With Radiation Mitigators Identifies Bacterial Associations With Survival”

**DOI:** 10.3389/fcimb.2021.715396

**Published:** 2021-09-21

**Authors:** Kelvin Li, Michael W. Epperly, Gabriella Acosta Barreto, Joel S. Greenberger, Barbara A. Methé

**Affiliations:** ^1^Center for Medicine and the Microbiome, University of Pittsburgh School of Medicine, Pittsburgh, PA, United States; ^2^Department of Radiation Oncology, University of Pittsburgh School of Medicine and UPMC Hillman Cancer Center, Pittsburgh, PA, United States; ^3^Division of Pulmonary, Allergy and Critical Care Medicine, Department of Medicine, University of Pittsburgh School of Medicine and UPMC, Pittsburgh, PA, United States

**Keywords:** microbiome, total body irradiation (TBI), Granulocyte Colony Stimulating Factor (G-CSF), JP4-039, necrostatin-1, baicalein, irritable bowel disease (IBD), Cox proportional hazards models

## Abstract

Total body irradiation (TBI) has been demonstrated to alter the intestinal microbiome, but the effects of successful small molecule ionizing radiation mitigators on the intestinal microbiome are not well-known. Our survival experiments examined the effects of anti-cell death radiation mitigators on and in conjunction with the host’s microbiota. Mice received 9.25 Gy TBI and then were administered radiation mitigators 24 hours later. Passed stool were collected pre-irradiation, then on days 1, 3, 5, 7, 10, 14, 21, and 30 post-irradiation for 16S rRNA gene (V4 region) sequencing. The Cox proportional hazards (CPH) model was fit with taxonomic composition (time varying covariates) and treatment as predictors. In the first experiment, mice were administered drugs for “granulocyte stimulation and anti-apoptosis” in four protocol combinations: JP4-039 (anti-apoptosis), granulocyte colony-stimulating factor (G-CSF, granulopoietic precursor cell stimulator), both mitigators, and control. Survival improved relative to control (30.0%) for G-CSF (80%, p-value = 0.025), G-CSF/JP4-039 (70%, p-value = 0.084), but not for JP4-039 (50.0%). In the second experiment, mice were administered mitigation drugs “inhibiting programmed cell death” pathways: JP4-039 (anti-apoptosis), necrostatin-1 (anti-necroptosis), and baicalein (anti-ferroptosis), in eight combinations. The survival of JP4-039/baicalein (60.0%, p-value = 0.010) and JP4-039/baicalein/necrostatin-1 (60.0%, p-value = 0.06) treatment combinations were significantly different from the control (26.7%). The JP4-039/necrostatin-1 (46.7%) and baicalein/necrostatin-1 (40.0%) and singlet treatment combinations (26.7%) were not significantly different from the control. Despite differences between the baseline microbiota compositions of the two experiments, consistent changes in composition after irradiation were found: *Lactobacillus* decreased post-irradiation, relative to baseline. By day 7, microbiota perturbations had incompletely reversed, and no drug-specific differences were identifiable. The CPH model identified *Lactobacillus* and members of *Ruminococcaceae*, including *Ruminococcus*, as protective and *Akkermansia* as deleterious. By day 30, the microbiota of surviving mice had not returned to baseline, but the differences between experiments suggest the resultant microbiota composition of the survivors are stochastic or batch specific in nature, rather than a requirement for survival. In conclusion, the study determined that key taxa identified in fecal samples, when applied towards the prediction of TBI survival, improves the survival model relative to treatment information alone.

## Introduction

There is an increasing interest in the role of the microbiome on the host’s recovery after exposure to total body irradiation (TBI). Specifically, identifying how the microbiome influences the lethal radiation dose and whether the effect of radiation mitigator agents (Rwigema et al., 2011; Huang et al., 2016; Steinman et al., 2018; Thermozier et al., 2020), which when delivered 24 hours or later after TBI exposure, increases survival (Rwigema et al., 2011). Classical radiation biology explanations for the effect of TBI on experimental animals and humans has relied on the understanding that bone marrow hematopoietic stem cells that survive irradiation can regenerate all hematopoietic lineages: erythropoiesis, myelopoiesis, thrombopoiesis, and lymphopoiesis. The failure of bone marrow recovery after TBI, manifested by the severity of hematopoietic syndrome, is dependent upon the dose of total body irradiation. The irradiated host can be rescued with bone marrow transplantation or the administration of hematopoietic growth factors (Rwigema et al., 2011). Lethal dose (LD_50/30_) for TBI is characterized by the death from marrow failure in 50% of subjects at 30 days. When radiation doses exceed the capacity of the intestinal crypt stem cells to regenerate the intestinal villi, manifested as gastrointestinal syndrome, the hosts cannot be rescued with bone marrow transplantation. These higher TBI doses result in irreparable intestinal damage.

In these higher dose TBI experiments, where intestinal damage has occurred and bone marrow directed treatments are insufficient to rescue the host, biomarkers are quantified to measure the extent of intestinal radiation damage. Intestinal barrier breakdown is measured with peripheral blood assays for bacterial endotoxin levels (Pendyala et al., 2012; Bischoff et al., 2014), an indication that intestinal microbes have been released into the circulatory system. In addition, low levels of serum citrulline (Jianfeng et al., 2005), a biomarker for functional enterocytes, has been used as a measure of intestinal barrier failure. Both biomarkers have been used to estimate the probability of recovery after high doses of TBI.

Towards measuring the specific taxonomic composition of bacteria in the gut, advancements of sophisticated 16S rRNA gene sequence analysis methods have facilitated modern studies of the changes in the gut microbiome after TBI. Studies indicate that the nature of these changes may be used to predict survival (Thermozier et al., 2020). Recent studies have demonstrated changes in both metabolism of bile salts and bacterial taxa in the intestine after varying doses of total body irradiation (Goudarzi et al., 2016). Whether there are intestinal microbiome predictors that are detectable soon after TBI which can predict survival is not known. Taxa associated as protective or contributing to inflammatory bowel disease (IBD), including Crohn’s Disease and ulcerative colitis, have been implicated and widely studied. Their role in pathogenesis or towards gut homeostasis suggests that the gut microbiome may not only be an indicator of gut health status, but also an important determinant of host survival after TBI. Research on germ-free (GF) mice has discovered that these mice were more resistant to lethal radiation enteritis than those mice conventionally raised (Crawford and Gordon, 2005). Observations such as these reveal the complexity of the host-microbiota interdependency, highlighting the importance of studies that examine the nature and extent of external perturbations, such as regimens directed towards the host or microbiota.

In the present study, we evaluated whether administration of cytokine or small molecule radiation mitigators at 24 hours after TBI altered the microbiota. We also evaluated whether specific alterations in the microbiota at specific times after TBI along with mitigator administration correlated with survival. We chose two experimental models to test the hypothesis that specific changes in the microbiota composition correlated with survival. In both experimental protocols, we collected fecal samples from mice prior to irradiation (day 0), and then on multiple time points up to thirty days after irradiation. Fecal samples, a proxy for distal gut contents, were analyzed for the relative abundance of bacterial taxa identified by 16S rRNA gene sequencing.

In the first model, “granulocyte stimulation and anti-apoptosis” (GSAA), we tested the well-known radiation mitigator, granulocyte-colony stimulating factor (G-CSF) (Satyamitra et al., 2017), which stimulates the proliferation of primitive bone marrow hematopoietic stem cells and expands the hematopoietic stem cells, as well as committed multilineage progenitors, and committed lineage cell compartments after irradiation (Satyamitra et al., 2017). We also administered a small molecule, JP4-039, the anti-apoptotic nitroxide targeted to the mitochondria by a peptide isostere of the hemi-gramicidin molecule (Rwigema et al., 2011). Twenty-four hours after the 9.25 Gy TBI dose, which is lethal to 50% of mice at 30 days (LD_50/30_), the effect of the radiation mitigator treatments (G-CSF, JP4-039, or both) on the mice were compared.

In the second model, “inhibiting programmed cell death” (IPCD), we tested the effect of each of three different radiation mitigators, which target three distinct irradiation-induced programmed cell death pathways: apoptosis, necroptosis, and ferroptosis (Thermozier et al., 2020) with the drugs: JP4-039 (Rwigema et al., 2011), necrostatin-1 (Huang et al., 2016; Steinman et al., 2018), and baicalein (Thermozier et al., 2020), respectively. These drugs were administered singly, in doublet protocols, or in a protocol with all three drugs. The optimized dose protocols used for the three agents were previously reported (Thermozier et al., 2020). The second model’s experiment was performed with a significantly larger sample size to accommodate the testing of additional mitigation drug regimens and to confirm the microbiota associations with survival that were identified in the first model’s experiment.

The results demonstrate a striking association between the abundance of *Lactobacillus*, and other specific taxa, with survival to 30 days based on the microbiota composition identified during the critical period of bone marrow hematopoietic cell regeneration between days 9-24. Our study provides evidence that predictability of survival post TBI improves when including information on the intestinal microbiota relative to controlling for the administration of radiation mitigator drugs alone. These results provide support for the potential effectiveness of new probiotics protocols that depend on strains of *Lactobacillus*, and also reveal candidate taxa to target for modulation as potential interventional strategies to improve TBI survival.

## Materials and Methods

### Delivery of Single or Multiple Mitigators to TBI Mice

#### GSAA (“Granulocyte Stimulation and Anti-Apoptosis”) Experiment: JP4-039 and G-CSF

Forty mice (N = 40) received 9.25 Gy TBI and 24 hours later received radiation mitigators. Mice were divided into 4 groups of n = 10: 1.) control, 2.) intramuscular JP4-039 (20 mg/kg, I.M.) 3.) subcutaneous G-CSF (0.68 mg/kg, S.Q.) (Satyamitra et al., 2017), and 4.) Both JP4-039 and G-CSF (See **Table 1**, “Experiments and Treatment Groups”).

**Table 1:** Experiments and treatment groups.

GSAA Experiment	N	IPCD Experiment	N
“granulocyte stimulation and anti-apoptosis”		“inhibiting programmed cell death”	
*Control:*	10	*Control:*	15
*Singlets:*		*Singlets:*	
JP4-039	10	JP4-039	15
G-CSF	10	baicalein	15
		necrostatin-1	15
*Doublet:*		*Doublets:*	
JP4-039 + GCSF	10	JP4-039 + baicalein	15
		baicalein + necrostatin-1	15
		JP4-039 + necrostatin-1	15
		*Triplet:*	
		JP4-039 + baicalein + necrostatin-1	15
**Total**	**40**	**Total**	**120**

This study consists of two experiments, GSAA and ICPD. The GSAA experiment were composed of 4 treatment groups of 10 mice each for a total of 40 subjects. The IPCD experiment was composed of 8 treatment groups of 15 mice each, for a total 120 subjects.

#### IPCD (“Inhibiting Programmed Cell Death”) Experiment: JP4-039, Baicalein, and Necrostatin-1

One hundred and twenty mice (N = 120) received 9.25 Gy TBI followed by mitigation treatment 24 hours later, with the exception of necrostatin-1 which was administered at 48 hours by itself, or at 72 hours when used in combination. (See **Table 1**, “Experiments and Treatment Groups”) There were 8 group/combinations of the 3 drugs, with each consisting of n = 15 mice: 1.) control, 2.) JP4-039 (20 mg/kg I.M., 24H), 3.) baicalein (50 mg/kg I.P., 24H), 4.) necrostatin-1 (1.65 mg/kg I.V., 48H), 5.) baicalein (24H) + necrostatin-1 (72H) 6.) JP4-039 (24H) + baicalein, 7.) JP4-039 (24H) + necrostatin-1 (72H), and 8.) JP4-039 (24H) + baicalein (24H) + necrostatin-1 (72H).

In both experiments, fecal samples were collected on days 0, 1, 3, 5, 7, 10, 14, 21 and 30 for 16S rRNA gene analyses. Day 0 samples were considered pre-irradiation (baseline). Day 1 samples were post-irradiation and pre-mitigation treatment. Day 7 samples were taken from all mice, prior to any sacrifices due to their development of hematopoietic syndrome. Radiation was delivered with a JL Shepherd Cesium Irradiator, Model 68.

### DNA Extraction

DNA extraction was performed using the Qiagen DNeasy Powersoil Kit (Qiagen, Germantown, MD) and processed per the manufacturer’s protocol. Reagent blanks were included as negative controls and cells from a microbial community of known composition (ZymoBiomics Microbial Community Standards; Zymo Research, Irvine, CA) as a positive control.

### Bacterial Community Sequencing

Extracted genomic DNA (gDNA) was amplified using Q5 HS High‐Fidelity polymerase (New England BioLabs, Ipswich, MA). Approximately 5 ng of each sample were amplified in 25 µL reactions. Cycle conditions were 98°C for 30 seconds, then 30 cycles of 98°C for 10 seconds, 57°C for 30 seconds, and 72°C for 30 seconds, with a final extension step of 72°C for 2 minutes. Triplicates were combined and purified with AMPure XP beads (Beckman Coulter, Indianapolis, IN) at a 0.8:1 ratio (beads:DNA) to remove primer dimers. Eluted DNA was quantitated on a Qubit fluorimeter (Life Technologies, Grand Island, NY). Sample pooling was performed on ice by combining 40 ng of each purified band. For negative controls and poorly performing samples, 20 µL of each sample was used. The sample pool was purified with the MinElute PCR purification kit (Qiagen, Germantown, MD). The final sample pool underwent 2 more purifications: AMPure XP beads to 0.8:1 to remove primer dimers, and a final cleanup in Purelink PCR Purification Kit (Life Technologies). The purified pool was quantitated in triplicate on the Qubit fluorimeter prior to sequencing.

The sequencing pool was prepared as per Illumina’s recommendations (Illumina, Inc., San Diego, CA), with an added incubation at 95°C for 2 minutes immediately following the initial dilution to 20pM. The pool was then diluted to a final concentration of 7pM + 20% PhiX control (Illumina). Sequencing was done on an Illumina MiSeq 500‐cycle V2 kit (Illumina).

### Bioinformatics

Sequences from the Illumina MiSeq were deconvolved and then processed through the Center for Medicine and the Microbiome (CMM) in‐house sequence quality control pipeline, which includes dust low complexity filtering, quality value (QV<30) trimming, and trimming of primers used for 16S rRNA gene amplification, and minimum read length filtering. Forward and reverse reads were merged into contigs then processed through the CMM’s Mothur‐based (Schloss et al., 2009) 16S rRNA gene sequence clustering and annotation pipeline. Sequence taxonomic classifications was performed with the Ribosomal Database Project’s (RDP) naïve Bayesian classifier (Wang et al., 2007; Quast et al., 2013) with the SILVA 16S rRNA database (v138) (Quast et al., 2013).

### Data Analysis

Due to the compositional nature of the taxonomic profiles from 16S rRNA gene sequencing (Gloor et al., 2017), taxonomic abundances were first transformed using the additive log ratio (alr) transformation (Tarabichi et al., 2015). The top 15 taxa, by average abundance across the experimental samples, were selected to represent the taxa of interest, and the remaining taxa were accumulated into the denominator of the ratio, prior to natural log transformation. Log ratio transformations are crucial when including multiple taxa into linear models to ensure the abundances are normally distributed and independent from each other (Aitchison, 1986).

Analyses involving the calculation of a diversity index utilized the Shannon diversity index and the Tail statistic (Li et al., 2012). The Tail statistic is more sensitive towards the lower abundance taxa than the Shannon diversity index. Analyses requiring the calculation of pair-wise compositional distances between samples used the Manhattan distance, which is also more sensitive towards differences in the lower abundance taxa than the Euclidean distance.

Paired analyses (Stapleton et al., 2020) for changes in taxonomic abundance, distance, and diversity were performed between specific time points from the same subject, while controlling for the effect of the treatment group. To examine the effect of irradiation on the microbiota, day 0 (baseline) was contrasted against day 1 (post-radiation, pre-treatment). To examine the effect of mitigation treatment on the post-irradiation microbiota, day 1 (post-radiation, pre-treatment) was contrasted against day 7 (last time point before any mice were sacrificed due to their development of hematopoietic syndrome). To examine whether an individual mouse microbiota had returned to their own baseline, day 0 (baseline) was contrasted against day 30 (last sample taken, and end of experimental observations). Since the response variable for the regression was the difference between the two time points, the estimated coefficient for the intercept represents the change for the control, and coefficients calculated for each group are relative to the control.

The Cox proportional hazards model was fit across the two experiments separately. The alr transformed taxonomic abundances were treated as time-varying covariates since multiple stool samples were taken per mouse. When the analysis was performed, the model utilized all the repeated measures of stool (days 0 to 30) that were available from each mouse. Radiation mitigation treatment drug combinations were considered static covariates since they were the experimental groups. The R hazard library was used to fit the model and estimate the hazard statistics (Therneau and Grambsch, 2000; Therneau and Lumley, 2014). For each experiment, two models were fit to evaluate the influence of the microbiota on the hazard rate. The full model included both the microbiota alr and mitigation drug treatments predictors, while the reduced model only included the treatment groups as predictors. The two experiments (GSAA and IPCD) were analyzed independently as they were performed separately, although the same protocols and practices were followed to ensure comparability between results.

Two linear models based on alternative codings of the treatment schemes were considered. The first model (Model 1) assumed treatments should be a linear combination of single drug main effects (e.g., A + B) and multiple drug interaction effects (e.g., A*B) terms. The second model (Model 2) assumed each drug combination was its own indivisible treatment plan, (e.g., A + B + C, where C is the A&B combined treatment). In the IPCD experiment, the resultant estimated coefficients of the main and interaction effects from the first model, confirmed that the improvement in survival for the combined treatments was not additive across the mitigation drugs that were included in them. The second linear model, which treated each drug combination as its own indivisible treatment plan, was more appropriate for interpreting the IPCD experiment, since at least two mitigation drugs (targeting the simultaneous inhibition of alternate cell death pathways) was necessary to be administrated for any improvement of survival. As a neutrophil stimulator, the G-CSF treatment in the GSAA experiment appeared to have at most a modest additive effect towards survival, suggesting that Model 1 was more appropriate. However, to facilitate the comparison of the two experiments, both experiments were analyzed with Model 2, since the benefit of modeling the larger and more complex IPCD experiment with Model 2 significantly outweighed any potential benefit of modeling the less complex GSAA experiment with Model 1.

## Results

### Survival

**Figure 1:**
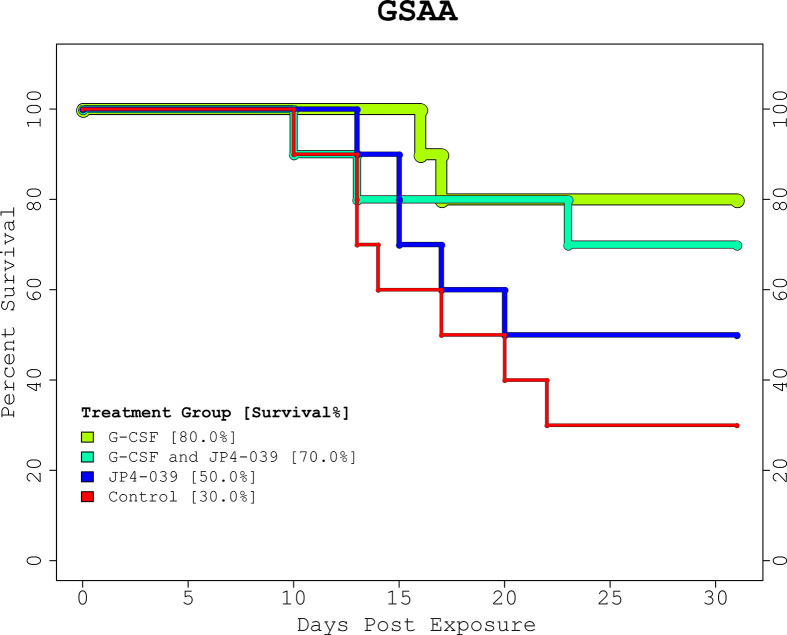
GSAA Experiment Kaplan-Meier Plot. The KM plot for the 2 drug GSAA experiment demonstrates the survival rate of the 4 treatment combinations. Day 0 and 1 are baseline and post-irradiation, respectively. Note the first necessary sacrifice occurs after day 9, and survivors past day 24 were assumed to have recovered from the TBI. Neither treatment nor control, guaranteed full survival or recovery. The treatment groups receiving G-CSF outperformed those with JP4-039 or control. The JP4-039 treatment was not statistically significantly different from the control with a p-value = 0.346.

**Figure 2:**
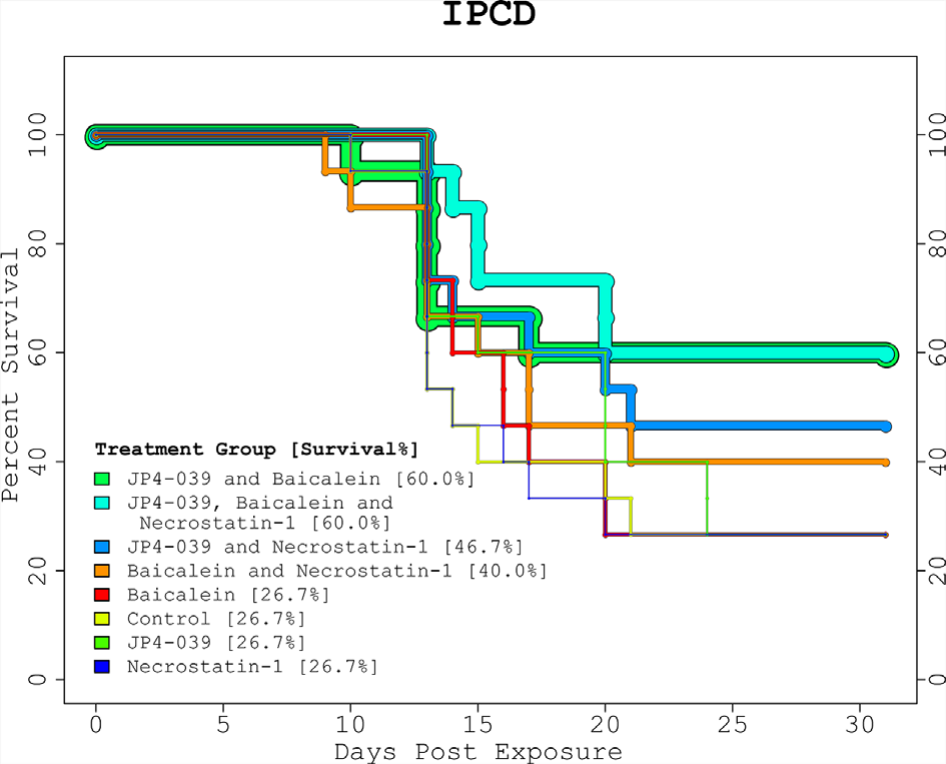
IPCD Experiment Kaplan-Meier Plot. The KM plot for the 3 drug IPCD experiment illustrates the survival rates of the 8 treatment combinations. Day 0 and 1 are baseline and post-irradiation, respectively. The critical period between days 9 to 24, when any mice requiring euthanasia were sacrificed, match the time period in the GSAA experiment. The single drug treatments performed as well as the control with a 26.67% survival rate. The top performing combinations included both JP4-039 and baicalein. With and without necrostatin-1 (p-value = 0.0359, and p-value = 0.0995, respectively), these treatment groups were statistically significantly different from the control, with survival rates of 60% *versus* 26.7%.

The proportions of survival from the GSAA experiment for control, JP4-039, G-CSF, and combined (JP4-039 + G-CSF), was 30%, 50%, 80%, and 70%, respectively (**Figure 1**, “GSAA: Kaplan-Meier Plot”). Both GCSF and combined, were statistically significantly different from control, with p-values of 0.0251 and 0.0839, respectively, as estimated with the log-rank test. JP4-039 by itself, was not considered a statistically significant improvement over the control (p-value = 0.346). The proportion of survival from the IPCD experiment for the single drug experiments (baicalein, JP4-039, and necrostatin-1), were the same as for the control at 26.67%. The baicalein/necrostatin-1, JP4-039/necrostatin-1, JP4-039/baicalein/necrostatin-1, and JP4-039/baicalein combinations, had survival rates of 40%, 46.67%, 60% and 60% respectively (**Figure 2**, “IPCD: Kaplan-Meier Plot”). According to the log-rank test, only the JP4-039/baicalein and JP4-039/baicalein/necrostatin-1 combinations were statistically significantly different from the control, with p-values of 0.0995 and 0.0359, respectively.

### The Average Taxonomic Composition

**Figure 3:**
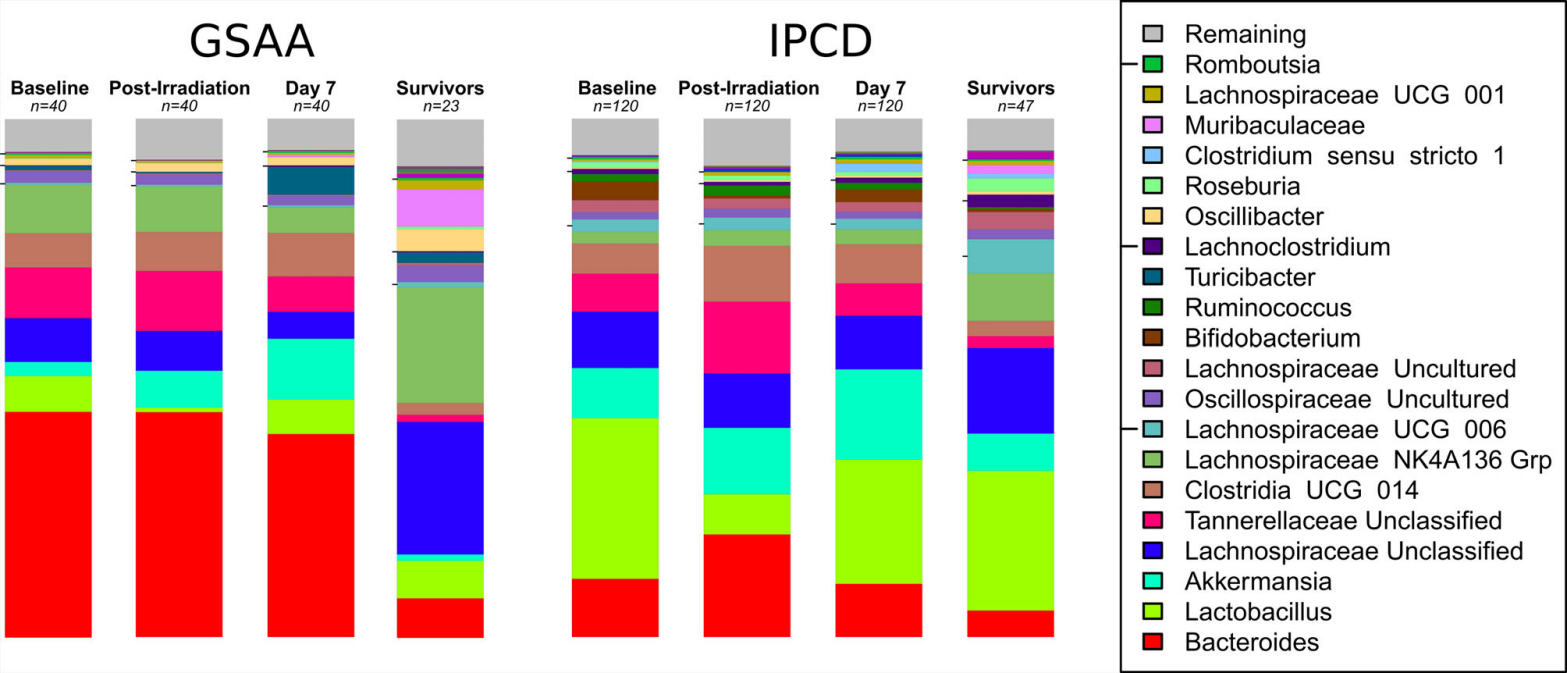
Stacked Bar Chart of Microbiota at Key Time Points. The stacked bar charts represent the average relative abundance at each time point of the top taxa between both experiments. Experiment GSAA and IPCD (left and right, respectively), each have 4 time points depicted: Baseline (Day 0), Post-Irradiation (Day 1), Post-treatment but before any sacrifices (Day 7), and Survivors (Day 30). The taxa are ordered by decreasing average abundance across all samples from bottom (*Bacteroides*) to the top (Remaining). Taxa in the Remaining category represent multiple taxa with abundances too low to individually represent visually. The baseline, post-irradiation, and day 7 consist of n = 40 and n = 120 samples, for the GSAA and IPCD experiments, respectively. The stacked bar chart for the survivors only consisted of the n = 23 and n = 47 samples from the GSAA and IPCD experiments, respectively, so survivor bias will be present here. From these charts, the difference in the baseline, i.e. batch specific differences, and their response to TBI can be qualitatively observed. *Lactobacillus* can be clearly seen to decrease in the post-irradiation samples, then recover by day 7. The taxonomic composition of the survivors have not returned to baseline.

The top 15 taxa identified in the GSAA experiment across all time points, by decreasing abundance were: *Bacteroides, Akkermansia, Lachnospiraceae NK4A136 Grp, Lachnospiraceae Unclassified, Tannerellaceae Unclassified, Clostridia UCG 014 ge, Lactobacillus, Turicibacter, Oscillibacter, Oscillospiraceae Uncultured, Muribaculaceae ge, Anaeroplasma, Colidextribacter, Ruminococcaceae Incertae sedis*, and *Lachnospiraceae UCG 001*. In the IPCD experiment, the following top 15 taxa (across all time points) were identified: *Lactobacillus, Akkermansia, Bacteroides, Lachnospiraceae Unclassified, Tannerellaceae Unclassified, Clostridia UCG 014 ge, Lachnospiraceae NK4A136 Grp, Lachnospiraceae UCG 006, Lachnospiraceae Uncultured, Bifidobacterium, Oscillospiraceae Uncultured, Ruminococcus, Lachnoclostridium, Clostridium sensu stricto 1*, and *Roseburia* (**Figure 3**, “Stacked Bar Chart of Microbiota at Key Time Points”).

### The Effect of Radiation on Microbiota (Day 0 *vs* Day 1, Baseline *vs.* Post-Irradiation)

**Figure 4:**
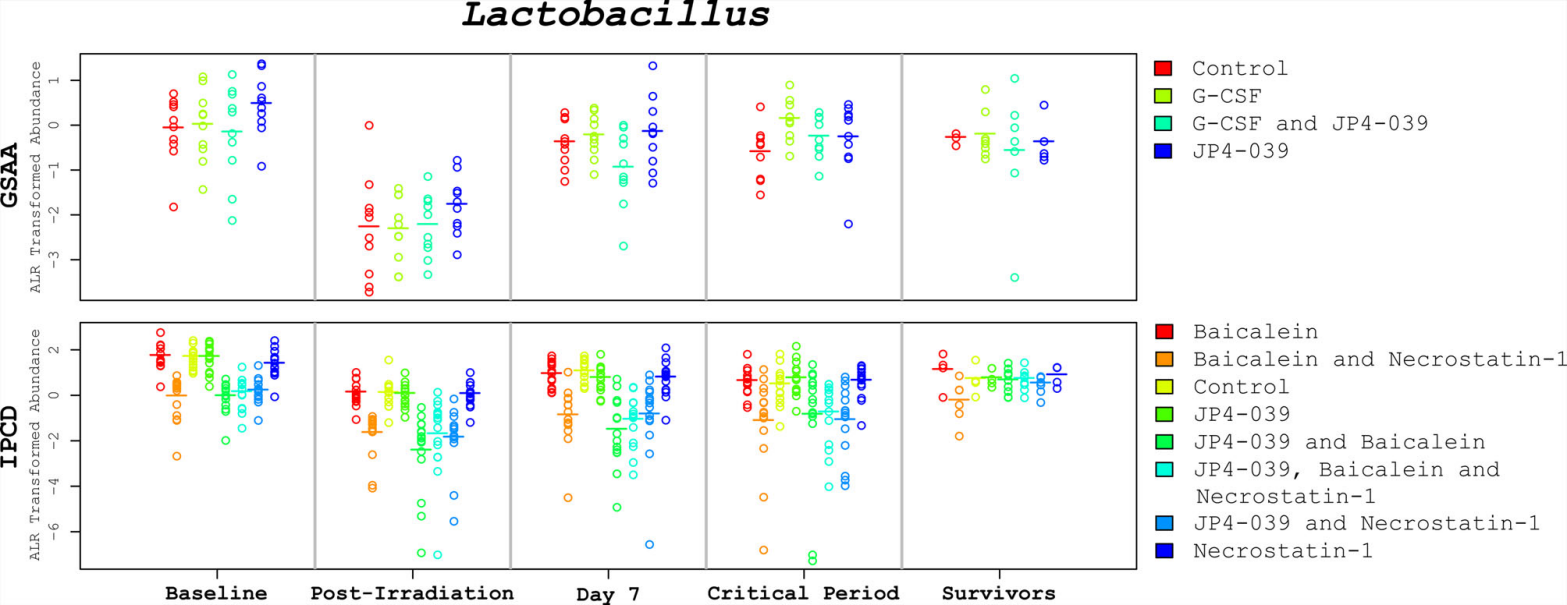
*Lactobacillus* abundances across the time points. These plots depict the change of *Lactobacillus* levels (alr transformed) across critical time periods. The GSAA and IPCD experimental results are positioned at the top and bottom, respectively. Each annular glyph represents an individual mouse and each horizontal bar represents the median for each treatment group. The order of the treatment groups from left to right, match the colors and order in the legend on the right margin, from top to bottom. The values represented in the critical period (days 9-24) are the average across the available samples for each mouse. These plots illustrate not only how the median *Lactobacillus* levels change between time points, but also the degree of variation within each treatment group. In spite of treatment group specific variation at baseline, the relative change of *Lactobacillus* appears to be conserved, suggesting that the observed changes are not simply an artifact of mean reversion.

*Diversity.* There were no statistically significant differences in diversity from the GSAA experiment after irradiation. In the IPCD experiment, there was an overall increase in Shannon diversity, with p-value < 0.0001, estimated with the Wilcoxon signed-rank test. Although no mitigation drug had been administered at this time point, the change in diversity was lower in the 4 groups of mice targeted for the combined drug treatments. This was likely due to cage effects. The overall increase in Shannon diversity was likely due to the decrease in *Lactobacillus*, since its relative abundance was reduced between time points. The Tail statistic did not identify a statistically significant difference between day 0 and day 1 using the Wilcoxon signed-rank test. Discrepancies between the findings of the Tail *versus* Shannon diversity index provide insight into where along the rank abundance curve the diversity has changed. In this case, diversity did not change significantly (**Figure 4**, “*Lactobacillus* abundances across the time points”).

*Composition.* In the GSAA experiment, there was a significant difference (coef = 0.454, p-value < 0.0001) in the overall composition between the day 1 and day 0 microbiota. There was a slightly greater difference in the JP4-039/G-CSF group over the control, but only marginally significant (coef = 0.170, p-value = 0.0936). In the IPCD experiment, there was also a significant change (coef = 0.944, p-value < 0.0001) in the composition post-irradiation. Again, the mice groups targeted for combined drugs, exhibited statistically significantly smaller changes (with coefficients ranging from -0.29 to -0.33 (less) than the control, with p-values ranging from 0.0009 to < 0.001).

*Abundance.* The GSAA experiment identified a statistically significant reduction in the abundance of *Lactobacillus* (coef = -2.21, p-value < 0.0001) and *Turicibacter* (coef = -2.43, p-value = 0.0001) post-irradiation. The IPCD experiment identified a broader number of taxa statistically significantly (p-values < 0.001) affected by irradiation. Taxa decreasing in abundance included: *Lactobacillus* (coef = -1.59, p-value < 0.0001), *Bifidobacterium* (coef = -2.88, p-value < 0.0001), and *Clostridium sensu stricto 1* (coef = -1.07, p-value = 0.0030). Taxa increasing in abundance included: *Akkermansia* (coef = 1.58, p-value < 0.0001), *Tannerellaceae Unclassified* (coef = 1.32, p-value < 0.0001), *Clostridia UCG 014* (coef = 0.95, p-value = 0.0020), and *Lachnospiraceae UCG 006* (coef = 0.82, p-value = 0.0023). There were also treatment group specific taxonomic differences (mostly decreases in abundance) that likely explained the previously acknowledged differences in diversity and composition (See **Supplemental Table 1**, “Paired-difference ALR Regression Coefficients for Day 0/Day 1”).

### The Effect of Treatment on Microbiota (Day 1 *vs.* Day 7, Post-Irradiation *vs.* Before First Sacrifice)

*Diversity.* In the GSAA experiment, only a marginally significant increase of diversity (intercept coef = 0.19, p-value = 0.098) was identified between day 1 post-irradiation and day 7 across all samples using the Shannon diversity index. The changes in diversity for the 3 treatments were not significantly different from the control. In the IPCD experiment, only the baicalein/necrostatin-1 group had a significant decrease of diversity (coef = -0.91, p-value = 0.0396), when measured by the Tail statistic. All other treatments were more similar to the control.

*Composition.* In the GSAA experiment, there was a statistically significant difference in the composition of the control group (intercept coef = 0.7419, p-value < 0.0001) between time points. The JP4-039 group had a smaller difference (coef = -0.1582, p-value = 0.0647) relative to the control. The changes in composition in the other treatments were not significantly different from the changes in the control. In the IPCD experiment, the changes in composition in the control (intercept coef = 0.7846, p-value < 0.0001) were similar in magnitude to the effects seen in the control group of the GSAA experiment. The baicalein (coef = 0.1781, p-value = 0.0145) and necrostatin-1 (coef = 0.1647, p-value = 0.0306) treatments had greater compositional differences between time points relative to the control. Change in composition for the other five treatment groups were not significantly different from the control.

*Abundance.* In the GSAA experiment, the abundances of *Lactobacillus* (coef = 1.874, p-value < 0.0001) and *Turicibacter* (coef = 5.487, p-value < 0.0001) increased, while abundance of *Lachnospiraceae NKA146 Grp* (coef = -0.876, p-value = 0.0057) decreased in the control. The composition in the G-CSF and JP4-039 treatments were not significantly (p-value > 0.05) different from the control. The combined JP4-039/G-CSF treatment identified increases in *Muribaculaceae* (coef = 3.450, p-value = 0.0003) and *Lachnospiraceae NK4A136 Grp* (coef = 1.050, p-value = 0.0175), and decreases in *Turicibacter* (coef = -2.285, p-value < 0.0048). [See **Supplemental Table 2**. “Paired-difference in taxonomic abundances between Post-Irradiation and Before First Sacrifice (Day 1 *vs* Day 7)”].

In the IPCD experiment, the abundances of *Lactobacillus* (coef = 1.357, p-value = 0.0002), *Bifidobacterium* (coef = 1.929, p-value = 0.0004), and *Clostridium senus stricto 1* (coef = 3.882, p-value < 0.0001) increased while *Tannerellaceae Unclassified* (coef = -0.767, p-value = 0.0015) decreased in the control. There were also modest treatment group specific differences, however they did not appear to be systematic. In the JP4-039/baicalein treatment group, an increase of *Bifidobacterium* (coef = 2.250, p-value = 0.0035) relative to the control was identified. In the necrostatin-1 treatment group, recovery of *Clostridium sensu stricto 1* was less than that of the control (coef = -2.373, p-value = 0.0084), although a cumulative increase in abundance (3.882 – 2.373 = 1.509) was identified. Other treatment group specific taxonomic changes not seen in the control included: baicalein (*Roseburia*, coef = 1.987, p-value = 0.0003), baicalein/necrostatin-1 (*Lachnospiraceae Uncultured*, coef = -1.631, p-value = 0.0034), JP4-039 (*Roseburia*, coef = 1.481, p-value = 0.0060), JP4-039/baicalein/necrostatin-1 (*Lachnospiraceae Unclassified*, coef = 0.704, p-value = 0.0098).

For both the GSAA and IPCD experiments, modest changes in the abundance of key taxa identified between day 0 and day 1, had reversed between day 1 and day 7. From these results, treatment apparently had little effect on the reversal of microbiota changes due to irradiation. Instead, reversal was more likely due to other biological factors. Despite this recovery, the microbiota had still not returned to baseline as the composition was still statistically significantly different between day 0 and day 7 for both the GSAA (coef = 0.6320, p-value < 0.0001) and IPCD experiments (coef = 0.6889, p-value < 0.0001) (**Figure 3**, “Stacked Bar Chart of Microbiota at Key Time Points”).

### The Association Between Microbiota and Survival

Using the Cox proportional hazards model, both experiments were analyzed first without the contribution of the microbiota (reduced model), then with the microbiota included (full model), so that improvements to the model fit could be discerned. Hazard ratios (HR) < 1 imply better survival, thus the closer the HR is to 1, the smaller the effect size of the factor on survival. An HR > 1 implies a deleterious contribution for that factor.

In the GSAA experiment, the G-CSF and JP4-039/G-CSF treatments showed statistically significant improvements in survival, with hazard ratios (HR) of 0.1918 [95% CI = (0.03968, 0.9268), p-value = 0.0399) and 0.3176 (95% CI = (0.08161, 1.2357), p-value = 0.0980], respectively. The JP4-039 treatment, showed a trend towards protectiveness, although not statistically significant (HR = 0.5877, 95% CI = (0.18631, 1.8542), p-value = 0.3646). The concordance (with values closer to 1 indicating better concordance) for the model fit was estimated to be 0.672.

When the fifteen most abundant taxa were included in the model (full model) as time varying covariates, the G-CSF treatment group showed an increase in effect size, accompanied by a reduction in significance [HR = 0.07281, 95% CI = (0.00379, 1.400), p-value = 0.0824]. The effect of the G-CSF/JP4-039 treatment was no longer significant and had a smaller effect size [HR = 0.57520, 95% CI = (0.05382, 6.147), p-value = 0.6473]. A protective effect was identified with the JP4-039 treatment [HR = 0.08922, 95% CI = (0.00741, 1.074), p-value = 0.0569]. Significant associations between microbiota and survival were identified. Taxa that were associated with better survivability were *Lactobacillus* [HR = 0.1490, 95% CI = (0.0463, 0.479), p-value = 0.0014], *Tannerellaceae Unclassified* [HR = 0.3247, 95% CI = (0.0928, 1.136), p-value = 0.0784], and *Ruminococcaceae Incertae sedis* [HR = 0.4724, 95% CI = (0.1974, 1.130), p-value = 0.0921]. In contrast, *Akkermansia* was associated with poorer survival [HR = 2.2387, 95% CI = (1.0141, 4.942), p-value = 0.0461]. Inclusion of taxa into the model increased model concordance to 0.911.

In the IPCD experiment, the reduced model alone identified only two combined treatments, JP4-039/baicalein and JP4-039/baicalein/necrostatin-1 with statistically significant HR < 1. Except for the necrostatin-1 treatment which had an estimated HR > 1 (p-value = 0.8884), the remaining treatments maintained a HR < 1 although the results were not statistically significant (p-values > 0.1819). The concordance of the reduced model was 0.606.

When the microbiota were included in the full model, only the triple drug combination of JP4-039/baicalein/necrostatin-1 had a marginally statistically significant improvement in survival [HR = 0.2847, 95% CI = (0.0656, 1.2353) p-value = 0.0933]. Each single drug treatment had a HR > 1, while each double drug combination had a HR < 1, none of these associations were statistically significant. Of the fifteen most abundant taxa included in the full model, four were associated with increased survival: *Lactobacillus* [HR = 0.6978, 95% CI = (0.5957, 0.8174), p-value = 8.29e−06], *Bifidobacterium* [HR = 0.7625, 95% CI = (0.6530, 0.8905), p-value = 0.000613], *Ruminococcus* [HR = 0.8512, 95% CI = (0.7290, 0.9938), p-value = 0.0415], and *Clostridium sensu stricto 1* [HR = 0.8136, 95% CI = (0.6920, 0.9566), p-value = 0.0125]. Three taxa were associated with decreased survival: *Tannerellaceae Unclassified* [HR = 1.7047, 95% CI = (1.1627, 2.4994), p-value = 0.006292], *Clostridia UCG 014 ge* [HR = 1.3075, 95% CI = (0.9713, 1.7601), p-value = 0.0771], and *Lachnospiraceae NK4A136 Grp* [HR = 1.2986, 95% CI = (1.0508, 1.6048), p-value = 0.0156]. The concordance of the full model was 0.861.

The addition of the microbiota to the Cox proportional hazards model improved model fit, based on the increase of model concordances from 0.672 to 0.911 (GSAA) and from 0.606 to 0.861 (IPCD). In addition, one would expect to see the effect and significance of the predictors (HRs) in the reduced model become diminished in a full model, if the addition of the extra predictors (taxa) to the full model were collinear with and stronger predictors than the subset of predictors (HRs) included in the reduced model. Interestingly, the estimated HR of each treatment groups in both experiments changed differently when the microbiota predictors were introduced into the model (See **Supplemental Table 4**. “Cox proportional hazards regression coefficients comparing experiments and models”). Since the treatments targeted different physiological pathways in the mouse, it follows that the composition of the microbiota could not simply be sufficient and necessary predictors. This would be the condition where survival could be predicted based on microbiota composition alone without foreknowledge of treatment, or if a specific microbiota composition by itself could be imposed as an independent radiation mitigator. These results support the presence of host-microbiota interactions. For example, if the mitigation drugs limited the homeostasis breakdown between the host and the microbiota, then the generation of any microbial byproducts crucial to the host’s recovery would not be interrupted and the deleterious effects of pathogenic microbiota on a TBI-induced heath-compromised host could also be limited.

Despite differences in the gut taxonomic composition identified from mice in the GSAA experiment *versus* the IPCD experiment, key microbiota identified as protective were consistently *Lactobacillus* and *Ruminococcus* (IPCD)/*Ruminoccocaceae* (GSAA), while the taxon that increased the hazard ratio was *Tannerellaceae Unclassified*. The taxon *Clostridia UCG 014* also had a HR > 1 in both experiments but was only marginally significant (p-value = 0.0771) in the IPCD experiment. Although *Bifidobacterium* was not identified among the fifteen most abundant taxa in the GSAA experiment, a significant protective association (p-value = 0.000613) was identified in the IPCD experiment.

The consistency between the survival models (GSAA and IPCD) from both experiments independently suggest that neither model was over fit due to the inclusion of excess taxa. Symptoms of overfitting linear models due to the inclusion of more predictors than can be supported by the available sample sizes, typically manifest themselves in the form of inconsistent findings between studies due to associations identified in one experiment being unidentifiable in another experiment. The key associations between microbiota and survival from the two experiments determined here are likely to be reproducible in future experiments, thus suggesting that these findings warrant further exploration.

### The State of Microbiota in Survivors (Day 0 *vs* Day 30, Baseline *vs.* Survivors)

*Diversity*. There was a statistically significant increase in diversity using both the Tail statistic (coef = 2.04, p-value = 0.0089) and the Shannon diversity index (coef = 0.43, p-value = 0.0297) in the GSAA experiment across the survivors. There were no treatment group specific differences found in the control group. In the IPCD experiment, a significant change in diversity was not identified using the Tail statistic in the control. In contrast, a reduction in diversity using the Tail statistic was identified in the baicalein group (coef = -2.27, p-value = 0.0481) and baicalein/necrostatin-1 group (coef = -2.47, p-value = 0.0199) relative to the control. The Shannon diversity did detect an increase in diversity in the control (coef = 0.33, p-value = 0.0447). Relative to the control, the baicalein/necrostatin-1 group (coef = -0.60, p-value = 0.0051) and JP4-039/baicalein/necrostatin-1 group (coef = -0.46, p-value = 0.0185) showed modest losses in diversity. A trend in lost diversity was also identified in the baicalein group (coef = -0.42, p-value = 0.0650).

**Figure 5:**
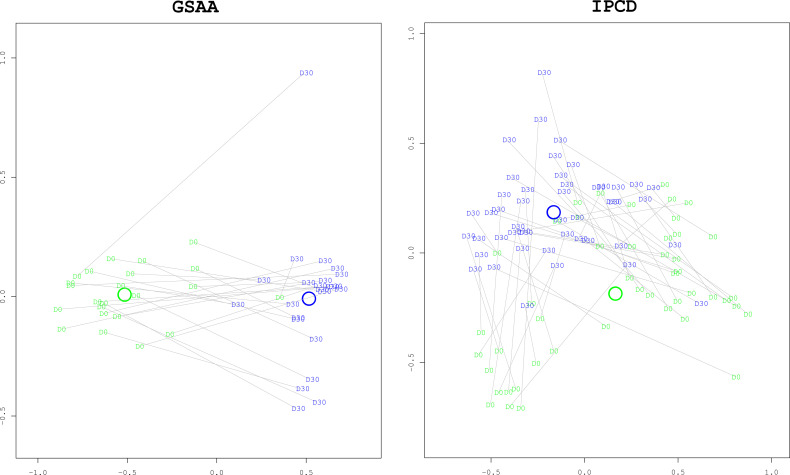
Multidimensional Scaling (MDS) plots comparing Baseline to Day 30 for Survivors. These ordination plots generated with the MDS algorithm illustrate that the overall composition of the survivors at day 30 (“D30”, blue) have changed significantly from baseline at day 0 (“D0”, green). The two dimensional (x and y axes) spatial relationships of the samples represent the degree of difference of taxonomic composition between the samples. Paired day 0 and day 30 samples from the same mouse are connected with grey lines. The x and y axes are unitless representations of the sample distance/dissimilarity estimated by the MDS algorithm. In both experiments, the difference in composition, as measured by the Manhattan distance, was statistically significant with bootstrapped p-values < 0.0001. As can be visually confirmed, the distance between the centroids of each group (represented by the green and blue annuli, representing day 0 and day 30, respectively) is greater than the dispersion within each group (the average distance between a group’s centroid and each of the members in the group).

*Composition*. The gut microbiota composition from the survivors in both the GSAA experiment (coef = 1.0334, p-value < 0.0001) and the IPCD experiment (coef = 1.0035, p-value < 0.0001) had changed significantly from baseline. In neither experiment were there any treatment group specific differences in their changes (**Figure 5**. “Multidimensional Scaling (MDS) Plot between Baseline and to Day 30 for Survivors”).

*Abundance*. In the GSAA experiment, there was a significant increase of *Muribaculaceae* (coef = 6.672, p-value < 0.0001) from baseline in the control group. A decrease in abundance of *Bacteroides* (coef = -2.315, p-value = 0.0239) relative to baseline was identified in the control. The abundances of *Muribaculaceae* and *Bacteroides* in the other treatment groups were not different from the control. The abundance of *Turicibacter* increased in the control (coef = 2.905, p-value = 0.039). The abundances of *Turicibacter* in the G-CSF and JP4-039 treatments were not different from the control but decreased in the JP4-039/G-CSF treatment (coef = -3.417, p-value = 0.0419 with an approximate net decrease of 2.905 – 3.417 = -0.512).

Other marginally significant changes (p-values between 0.05 – 0.10) in the control included less *Tannerellaceae* and *Anaeroplasma*, with no significant difference in the treatment groups. The G-CSF and JP4-039/G-CSF group showed decreases in *Akkermansia*. A decrease in *Clostridia UCG 014* was also identified in the JP4-039/G-CSF group. Nonetheless, these taxa with marginally significant changes from baseline were still generally important contributors in the Cox proportional hazard analysis. Thus, it is possible that with a larger experimental sample size, statistical significance would have been achieved.

In the IPCD experiment, the control group showed a decreased abundance of *Bacteroides* (coef = -1.964, p-value = 0.0027) and *Bifidobacterium* (coef = -3.063, p-value = 0.0035). Changes in abundance of *Bacteroides* in the treatment groups were not found. The abundance of *Bifidobacterium* increased in the JP4-039/baicalein/necrostatin-1 (coef = 3.857, p-value = 0.0086) and JP4-039/necrostatin-1 (coef = 4.515, p-value = 0.0036) groups relative to their baseline. The abundance of *Clostridium sensu stricto 1* increased (coef = 3.982, p-value = 0.0058) only in the JP4-039/baicalein treatment group. The *Lachnospiraceae uncultured*, decreased in the JP4-039/baicalein/necrostatin-1 (coef = -1.473, p-value = 0.0064) treatment group, but increased in the necrostatin-1 (coef = 1.651, p-value = 0.0091) treatment group. With p-values < 0.05, *Lactobacillus* abundance increased in the JP4-039/baicalein, JP4-039/baicalein/necrostatin-1, and JP4-039/necrostatin-1 treatment groups. The *Tannerellaceae Unclassified* abundance decreased while *Clostridium sensu stricto 1* abundance increased in the necrotstatin-1 group. There were other marginally significant (p-values 0.05 - 0.10) changes in *Lachnospiraceae* across the treatment groups, but they were not systematic. The complete table of p-values and coefficients is included in (See **Supplemental Table 3**. “Paired-difference in taxonomic abundances between Baseline and Day 30 of Survivors”).

## Discussion

Understanding the role of the gut microbiota in the context of TBI and the use of TBI-drug mitigators is an important and ongoing research challenge. There has been extensive research on the potential interactions between the microbiota and the gut of TBI subjects. For example, Goudarzi et al. (2016) (Goudarzi et al., 2016), focused on identifying gut taxonomic and metabolomics biomarkers from the stool of mice (N = 21) mice split evenly across three radiation doses (0, 5, and 12 Gy) with collected fecal samples at four time points (-1, 3, 14, and 30 days post-irradiation). The study identified significant changes in the microbiota and microbial-derived products. In particular, increased abundances of *Lactobacillaceae* and *Staphylococcaceae*, and decreased abundances of *Lachnospiraceae*, *Ruminococcaceae*, and *Clostridiaceae* were identified after irradiation. However, to preserve their sample size remaining by the end of their study, their chosen radiation dosages were not ideal for estimating the potential effects of specific taxa on mice survival. More recently, Guo et al. (2020) (Guo et al., 2020), also studied the effects of TBI on mice over time by irradiating (N = 57) mice and collecting fecal samples at five time points (-1, 2, 7, 21, and 30 days post-irradiation). They also found higher abundances of *Lachnospiraceae* and *Enterococcaceae* in the surviving mice.

The role of gut microbiota in conjunction with TBI-drug mitigators is less well understood. Our present study utilized a combined N = 40 (GSAA) + 120 (IPCD) = 160 mice, with fecal samples collected at nine time points (0, 1, 3, 5, 7, 10, 14, 21 and 30 days post-irradiation) while evaluating the effect of four mitigation drugs through ten unique combinations (including control). Compared to the prior studies, the greater sample size and sampling frequency especially during a critical period (days 9-24, when mice were recovering their hematopoietic cell production), provided an opportunity to study the relationship between the host, influenced by mitigation drugs targeting various immunological pathways, and its microbiota with a statistical survival model.

The results of the two experiments, the first (GSAA) testing GCSF and JP4-039 which are cytokine and small molecule radiation mitigators, and the second (IPCD) testing drug mitigators of apoptosis, necroptosis, and ferroptosis pathways, were largely consistent with each other in several important aspects. First, both experiments revealed the potential influence of the microbiota on the survival of mice and therefore supports the growing body of evidence that the gut microbiome plays an important role in host survival after TBI. In both experiments, inclusion of the relative abundance of dominant taxa as time varying covariates into the Cox proportional hazards model improved model concordance when compared to the (reduced) model with treatment alone. These results suggest that the influence of the microbiota on, or in response to, improved survival is crucially associated with host health. While the hazard model can only identify associations (not cause or effect), as an observational tool, it provides a useful filter for identifying taxa of interest for further study. Second, both experimental analyses identified key microbial taxa, with respect to their relative statistical significance of association, and their tendencies for protectiveness (HR<1), or deleteriousness (HR>1). Although the two experiments presented here were performed at different times, with different batches of mice and focused on different mitigation treatments, the resulting taxonomic associations were largely identical. The key taxonomic associations with hazard were largely consistent between experiments even though the overall taxonomic compositions of the distal gut microbiota were different between experiments at baseline (day 0). These results support the use of our statistical approach as a valid strategy for modeling the relationship between microbiota compositional data with host survivorship under different conditions (e.g., drug treatment regimens) over time.

Both experiments identified the presence of *Lactobacillus* and members of *Ruminococcaceae*, including *Ruminococcus*, as positive predictors of increased survival (**Supplemental Table 4**, “Cox proportional hazards regression coefficients comparing experiments and models”). Consistent with these findings, in a study of TBI in large mammals (macaques and Göttingen minipigs) *Lactobacillus* was significantly associated with radiation intensity and *Ruminoccoccus* maintenance over time was strongly associated with survival in the minipig model (Carbonero et al., 2019). These results suggest that findings in our study may scale to humans.

*Lactobacillus* has been identified as a largely beneficial commensal member of the gut that may confer beneficial properties to the host through multiple roles. These include stimulation of anti-inflammatory immune system mechanisms, enhancement of gut epithelial integrity through nuclear factor-κB and mitogen-activated protein kinase pathways (Lebeer et al., 2008), and antimicrobial activity towards potentially pathogenic microbes. In a recent study, inorganic nitrate supplementation through nitric oxide formation was determined to reduce TBI-induced colon injury in mice (Wang et al., 2020). *Lactobacillus* was found to be elevated in gut microbiota in mice at seven and thirty days after TBI in the nitrate supplemented group. Although further study is warranted, these results suggest that *Lactobacillus* could also influence the nitrate/nitrite/NO pathway through nitrate reduction or environmental acidification by lactic acid production facilitating reduction of nitrite to nitric oxide through non-enzymatic disproportionation (Tiso and Schechter, 2015).

The *Ruminococcaceae* family are members of the Firmicutes phylum that contain a range of organisms including *Ruminococcus* and other genera frequently identified in the mammalian gut microbiota such as *Faecalibacterium* and *Subdoligranulum*. In the gut, *Ruminococcaceae* are generally considered to be anaerobic and contribute to complex carbohydrate metabolism and production of short chain fatty acids (SCFAs) through fermentation. The balance of SCFAs is increasingly the subject of intense investigation for their roles in human metabolic health including control of intestinal inflammation (Byrne et al., 2015; Sanna et al., 2019).

A complex host-microbiota interplay exists between host production of liver-derived primary bile acids (PBAs), cholic acid, and chenodeoxycholic acid and intestinal bacteria which transform them into secondary bile acids (SBAs) (Pols et al., 2011; Schaap et al., 2014; Sinha et al., 2020). Multiple taxa (including *Lactobacillus*) possess bile salt hydrolases capable of deconjugating glycine and taurine residues from host-derived PBAs. However, taxa affiliated with the *Ruminococcaceae* family have also been shown to be among the limited bacterial members capable of the second biotransformation of these deconjugated bile acids by a 7a-dehydroxylation reaction to produce the SBAs deoxycholic acid and lithocholic acid. Microbially-generated SBAs are powerful signaling molecules critical to the regulation of colonic inflammatory processes. SBAs have been identified as risk factors in colon inflammation and cancer. For example, the reduction in 7a-dehydroxylating bacteria, including *Ruminococcaceae*, was associated with decreased SBAs and in increased intestinal inflammation in colectomy treated patients with ulcerative colitis (Sinha et al., 2020).

Additional taxa were also identified (Carbonero et al., 2019) in this study as either statistically significant or having similar trends as either positive or negative predictors of survival in both experiments. These organisms include negative associations with survival for *Akkermansia* and *Clostridia UCG 014* and positive associations with survival for *Tannerellaceae*, and *Bifidobacterium.* Each taxon has been identified in the mammalian gut, including humans, and have been associated with a variety of ecological roles in their hosts that range from opportunistic pathogens to commensal and mutualistic relationships. The best studied member of the genus *Akkermansia*, *Akkermansia muciniphila*, physically associates with the host intestinal mucus layer where it degrades mucin to obtain carbon and energy sources, with subsequent release of metabolites such as SCFAs, which can further be used as sources for metabolism such as fermentation by other members of the microbiome and they can impact host signal transduction pathways central to the maintenance of intestinal barrier integrity. As such, *Akkermansia* has been associated with health improvement (Derrien et al., 2017) and potentially negative impacts including associations with colorectal cancer in humans and as a trigger of colitis in a murine model (Seregin et al., 2017). *Clostridia* species are ubiquitous members of the gut that can function metabolically in fermentative processes that include SCFA production (Macfarlane and Gibson, 1995; Macfarlane and Macfarlane, 2003) and urobilinoid oxidation (Vítek et al., 2006), and act as opportunistic pathogens. *Tannerellaceae* (which include the genera *Tannerella* and *Parabacteroides*) have been associated with metabolic activation by flavonoids. The *Bifidobacterium*, members of the Actinobacteria phylum and lactic acid producing bacteria, share similar functional roles with *Lactobacillus*. Collectively, these results emphasize the presence of a polymicrobial gut community in subjects that can be influenced by TBI, and suggest a complex set of metabolic, physiological, and immune-mediated interactions (Riva et al., 2020; Osman et al., 2021) can occur between the host and gut microbiota. These interactions could influence host survival after TBI insult.

**Figure 6:**
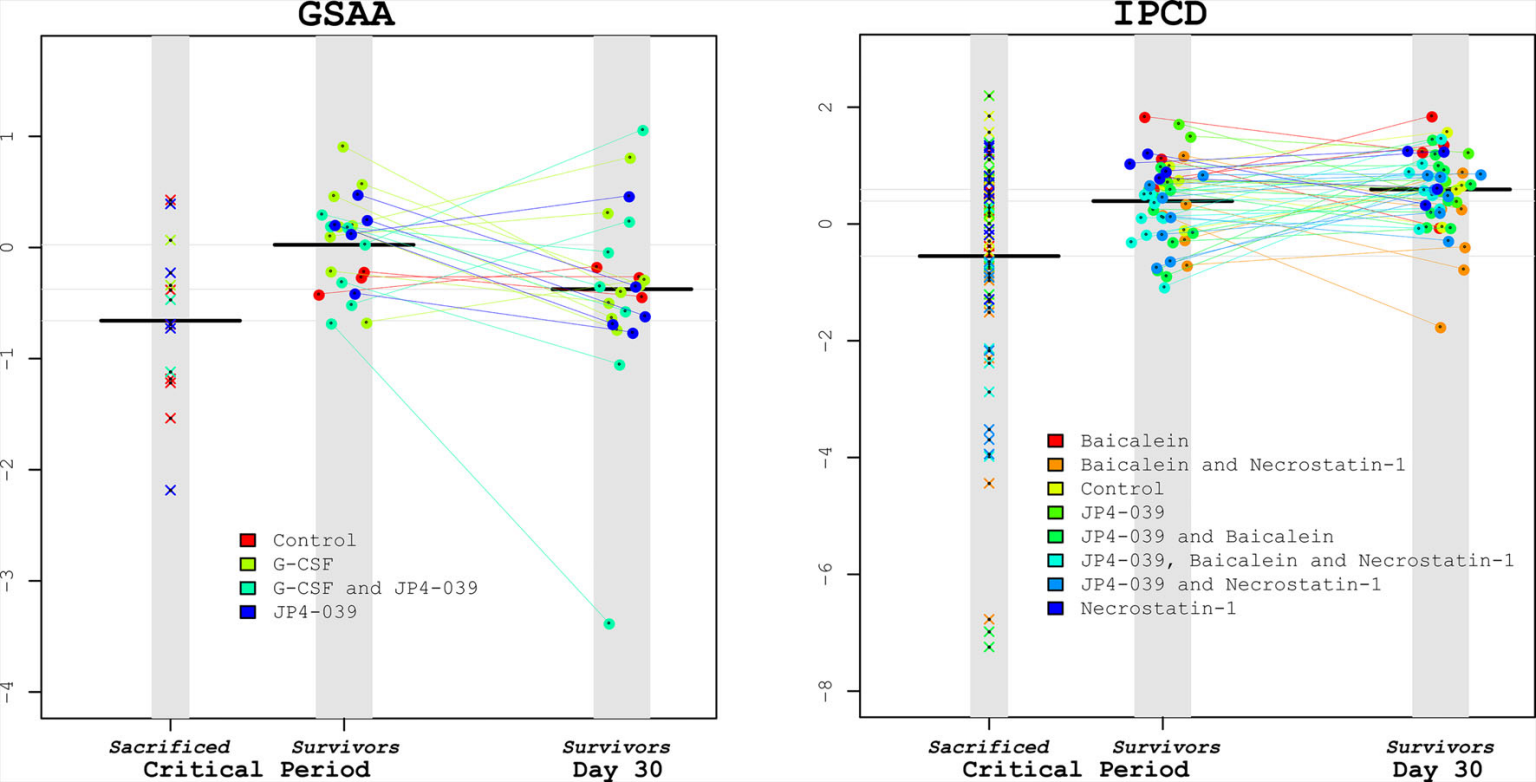
*Lactobacillus* levels comparing Sacrificed *vs.* Survivors. To visually illustrate the results from the survival analysis, *Lactobacillus* levels during the critical period were compared between the sacrificed *vs.* the surviving mice for the GSAA and IPCD experiments (left and right plots, respectively). Within each plot there are 3 columns: The left-most columns depict the average *Lactobacillus* levels from the samples available for the mice during the critical period that were eventually sacrificed. The center column depicts the *Lactobacillus* levels during the critical period for eventual surviving mice, connected with lines to the *Lactobacillus* levels on day 30 in the right-most column. The black horizontal bars represent the median *Lactobacillus* levels for each column. From these plots it can be confirmed that the median *Lactobacillus* levels of the sacrificed mice were lower than the survivors during the critical period for both experiments. The *Lactobacillus* levels of the eventual survivors were not significantly different between this critical period and at Day 30. These scatter plots illustrate the wide variance of the *Lactobacillus* levels in the sacrificed mice, suggesting that an intervention of supplemental *Lactobacillus* by itself may have limited benefits.

An important question to pose in this study is the extent to which microbiota taxonomic profiles can be used to predict survival in the context of the drug treatment regimens. After identifying that *Lactobacillus* was statistically significantly associated with improving survival (reducing the hazard rate) of the irradiated mice, would this knowledge be useful in predicting the survival of specific mice based on the assayed *Lactobacillus* levels and treatment alone? An equally important question is how far in advance would this knowledge prove informative? The closer to death a mouse is before the abundance of *Lactobacillus* is predictive, may suggest that a drop in *Lactobacillus* (relative) abundance is only a marker for host decline, rather than a protective agent. Based on the GSAA experiment, when the median abundances of *Lactobacillus* were compared between non-survivors and survivors at various time points, the median non-survivors were consistently lower than the median survivors. However, in the IPCD experiment, the median *Lactobacillus* levels were greater in the non-survivors during baseline, after irradiation, and before the first necessary sacrifice (day 7). Only when mice began to succumb to TBI between day 9 and day 24 (during the recovery of hematopoietic cell production), did the median *Lactobacillus* abundance in the survivors become greater than the non-survivors (**Figure 6**. “Lactobacillus levels of Survivors *vs.* Non survivors during days 9-24”). These results suggest interventions that solely boost *Lactobacillus* abundance before the critical period (before day 9) may be of limited value. Instead, strategies that maintain the polymicrobial nature of the healthy gut to preserve anti-inflammatory and other metabolic processes may prove more successful. Given the extensive literature supporting the importance of *Lactobacillus* towards host gut homeostasis, these results support the perspective that a completely host-centric approach toward treatment is likely to be short-sighted.

The mitigation drug treatments did not appear to have significant direct effect on the microbiota. In both experiments, there were statistically significant changes in composition from day 1 to day 7 in the control. In contrast, few significant increases or decreases in abundant taxa were identified in the treatment groups relative to the control. Further, the combined drug treatments were not correlated with, nor were they linear combinations of the individual drug treatments from which they were composed. For example, in the IPCD experiment, there was an increase in the abundance of *Roseburia* in the baicalein and JP4-039 treatment groups, but no difference in *Roseburia* abundance was identified for any of the combined treatments that included baicalein or JP4-039. The mitigation treatments were administered through different routes [intramuscularly (JP4-039), intraperitoneally (G-CSF), and intravenously (baicalein and necrostatin-1)] and not directly into the gut. Therefore, the gut microbiota were less likely to experience significant direct exposure to the drugs and instead were more likely impacted by a systemic host response to the drug treatment.

Examination of microbiota composition of the surviving mice at day 30 revealed that changes in overall composition was sufficient to differentiate them from their baseline profiles. However, the majority of abundant taxa when scrutinized individually were not statistically significantly different from baseline. At day 30, the most significant changes in the GSAA experiment showed an increased abundance of *Muribaculaceae* and in the IPCD experiment, decreased abundance of *Bacteroides* and *Bifidobacterium*. In the IPCD experiment, the decrease in *Bifidobacterium* was lower than the control for the JP4-039/baicalein/necrostatin-1 (60% survival) and JP4-039/necrostatin-1 (46.67% survival) combinations, which had the greater proportion of survivors. The JP4-039/baicalein treatment, which had a similarly high survival as the JP4-039/baicalein/necrostatin-1 treatment (60%), only exhibited differences from baseline with an increase in *Clostridium sensu stricto 1*. From these experiments, it is difficult to project whether these treatment specific differences would eventually disappear if observed weeks later without intervention, or if the changes were only non-consequential and stochastic differences due to batch specific variation in the mice. These results may also indicate that the biological functions and ecological roles undertaken by the microbiota are more important than specific composition to host recovery.

Evidence for batch specific microbiota changes was already identifiable between baseline and post-irradiated mice, since some treatment groups had already exhibited differences in their microbiota before any treatment had been administered. These findings underline the precariousness of only focusing on the changes between baseline and day 30 among the survivors. Survivor bias is an effect which should be considered when interpreting the results of survival-based studies. The Cox proportional hazards model, as applied in this study, models the association between survival rate and taxa. This approach provides a more robust statistical model as it is capable of simultaneously controlling for the treatment types and most abundant taxa that have been sampled multiple times from the same subject (repeated measures) and considers all subjects and their date of sacrifice.

Finally, results from this study may provide insights into host-microbiota interactions that occur in other diseases underlain by inflammatory mechanisms and imbalances in cell death pathways. For example, IBD and its two subtypes, ulcerative colitis and Crohn disease, are diseases of the gastrointestinal tract thought to result from interactions between immune-mediated processes, host genetic factors, environmental exposures and the microbiome. Although IBD is a chronic disease, whereas acute exposure to radiation is an insult to radiation sensitive cells throughout the body including bone marrow and the intestinal tract, there are nonetheless aspects of disease course that are similar. Subjects with IBD and the GI syndrome of TBI both suffer from the imbalance and increase in cell death of intestinal epithelial cells through cell death pathways (Günther et al., 2013; Xu et al., 2020). The baicalein (Jang et al., 2019; Liang et al., 2019), necrostain-1 (Liu et al., 2015) and GCSF (Meshkibaf et al., 2016) treatments have all been shown to have a mitigating effect on intestinal inflammation, although the additional production of neutrophils stimulated by G-CSF may increase the numbers recruited to the injury site to help fend off bacterial infiltration and bacteremia. Extensive studies of host-microbiota interactions in IBD models support a critical role of the microbiome in inflammatory processes. Changes in the abundance of specific taxa identified in the TBI experiments through the Cox proportional hazards model in this work, implicate taxa also associated with IBD, depending on disease model (Seregin et al., 2017; Hall et al., 2017; Henke et al., 2019; She et al., 2020). These findings suggest probable biological and ecological roles of these microbiota, that may include changes in SCFA and SBA composition and concentrations, could be affecting host recovery. These results may also suggest that the GI syndrome component of acute radiation syndrome, including gut microbiota interactions with the host especially inflammation status, can be further examined for additional parallels and intersections with IBD. A further advantage of a TBI-induced inflammation model for comparison with IBD is that the initiation or severity (dosage) of the insult triggering an inflammatory response can be withdrawn and controlled.

We recognize several limitations in our study. Our study was not designed to address mechanistic relationships between the murine host and microbiome. 16S rRNA gene analyses can only provide taxonomic units that are generally accepted as reliably differentiable at the genus level. However, pathogenic and commensal species and strains often co-exist within the same genus, and pathways for common metabolites are possessed by organisms independent of their distinct taxonomic classifications. Thus, multi-omics approaches such as whole community DNA (metagenomics) and RNA (metatranscriptomics) sequencing and metabolomics, would elucidate present and active metabolic and regulatory pathways and metabolites, respectively. Further, metagenomics and metatranscriptomics also provide expanded taxonomic profiles beyond the Domain Bacteria including Archaea and Fungi. For example, in the gut, the bacterial microbiota can play a role towards the suppression of fungal pathogens, such as *Candida*. As such, the bacterial community assayed by 16S rRNA gene sequencing alone may not provide sufficient information as a proxy for the degree of fungal suppression (Cabral et al., 2018). In addition, batch effects, also reported in other animal studies, could be a confounding factor in our two experiments, although we have shown that to some degree the results were consistent despite their presence.

Nonetheless, based on current promising results, additional future research is warranted towards further elucidating the effects of the microbiota on TBI survival. Additional work could also include testing additional supportive interventions such as probiotics, microbial transplants, and anti-inflammatory bacterial end products. The association between higher abundances of *Lactobacillus* and TBI survival support the possibility that second generation probiotics depending on *Lactobacillus* species, such the IL-22 producing recombinant *L. reuteri* (Zhang et al., 2020), may prove safe and effective, although identifying the precise conditions for supplement administration may be crucial for their efficacy.

In conclusion, experiments testing TBI mitigation drugs presented here have shown that examination of the distal gut microbiota can be informative towards predicting the survival of mice that have received TBI with 9.25 Gy. The taxa identified as associated with increased or decreased survival rates were replicated between experiments, even with different initial microbiota baselines and differing subsequent mitigation treatment regimens, suggesting that the taxonomic associations may have important biological and ecological implications. In particular, the maintenance of *Lactobacillus* and *Ruminococcaceae* abundances were consistently identified as significant to increased survival rates. The role of the microbiota was most influential during the critical period between day 9 and day 24, when there is recovery of hematopoietic cell production. There is limited evidence that the mitigation treatments had a direct effect on the microbiota. Instead, interactions between the microbiota and host in conjunction with mitigation treatments combined to limit the impact of the gastrointestinal syndrome, whose resolution was crucial for the irradiated mice to survive past a critical period of mortality. Taxa identified with survivorship in this study have been associated with metabolic properties that may influence inflammation and immune status. Collectively, these results provide the framework for additional future research towards further elucidating the effect of the microbiota on TBI survival including the identification of supportive interventions such as probiotics, or microbiota transplants.

## Data Availability Statement

The original contributions presented in the study are publicly available in NCBI using accession number PRJNA733204.

## Ethics Statement

The animal study was reviewed and approved by the University of Pittsburgh Institutional Animal Care and Use Committee under protocol number 18022000. The University of Pittsburgh animal facilities have been accredited by AALAC.

## Author Contributions

KL analyzed data and drafted the manuscript. ME designed and performed the experiment, collected the samples, and edited the manuscript. GA performed laboratory processing and generated data. JG conceived and designed the study, supervised the project, and edited the manuscript. BM generated and analyzed data and drafted the manuscript. All authors contributed to the article and approved the submitted version.

## Funding

Supported by grant from the NIH/NIAID U19A1068021, and the UPCI-Hillman Animal Research Core Facility award P30CA047904.

## Conflict of Interest

The authors declare that the research was conducted in the absence of any commercial or financial relationships that could be construed as a potential conflict of interest.

## Publisher’s Note

All claims expressed in this article are solely those of the authors and do not necessarily represent those of their affiliated organizations, or those of the publisher, the editors and the reviewers. Any product that may be evaluated in this article, or claim that may be made by its manufacturer, is not guaranteed or endorsed by the publisher.

## References

[B1] Atchinson J. (1986). The Statistical Analysis of Compositional Data (Monographs on Statistical and Applied Probability, J. R. Stat. Soc 44 (2), 139–60. doi: 10.1111/j.2517-6161.1982.tb01195.x

[B2] Bischoff S. C. Barbara G. Buurman W. Ockhuizen T. Schulzke J.-D. Serino M.. (2014). Intestinal Permeability–A New Target for Disease Prevention and Therapy. BMC Gastroenterol. 14 (1), 1–25. doi: 10.1186/s12876-014-0189-7 25407511 PMC4253991

[B3] Byrne C. Chambers E. Morrison D. Frost G. (2015). The Role of Short Chain Fatty Acids in Appetite Regulation and Energy Homeostasis. Int. J. Obes. 39 (9), 1331–1338. doi: 10.1038/ijo.2015.84 PMC4564526 25971927

[B4] Cabral D. J. Penumutchu S. Norris C. Morones-Ramirez J. R. Belenky P. (2018). Microbial Competition Between *Escherichia Coli* and *Candida Albicans* Reveals a Soluble Fungicidal Factor. Microbial Cell 5 (5), 249. doi: 10.15698/mic2018.05.631 29796389 PMC5961918

[B5] Carbonero F. Mayta A. Bolea M. Yu J.-Z. Lindeblad M. Lyubimov A.. (2019). Specific Members of the Gut Microbiota Are Reliable Biomarkers of Irradiation Intensity and Lethality in Large Animal Models of Human Health. Radiat. Res. 191 (1), 107–121. doi: 10.1667/RR14975.1 30430918

[B6] Crawford P. A. Gordon J. I. (2005). Microbial Regulation of Intestinal Radiosensitivity. Proc. Natl. Acad. Sci. 102 (37), 13254–13259. doi: 10.1073/pnas.0504830102 16129828 PMC1193536

[B7] Derrien M. Belzer C. de Vos W. M. (2017). Akkermansia Muciniphila and Its Role in Regulating Host Functions. Microb. Pathog. 106, 171–181. doi: 10.1016/j.micpath.2016.02.005 26875998

[B8] Gloor G. B. Macklaim J. M. Pawlowsky-Glahn V. Egozcue J. J. (2017). Microbiome Datasets Are Compositional: And This Is Not Optional. Front. Microbiol. Mini Rev. 8, 2224. doi: 10.3389/fmicb.2017.02224 PMC5695134 29187837

[B9] Goudarzi M. Mak T. D. Jacobs J. P. Moon B. H. Strawn S. J. Braun J.. (2016). An Integrated Multi-Omic Approach to Assess Radiation Injury on the Host-Microbiome Axis. Radiat. Res. 186 (3), 219–234. doi: 10.1667/RR14306.1 27512828 PMC5304359

[B10] Günther C. Neumann H. Neurath M. F. Becker C. (2013). Apoptosis, Necrosis and Necroptosis: Cell Death Regulation in the Intestinal Epithelium. Gut 62 (7), 1062–1071. doi: 10.1136/gutjnl-2011-301364 22689519

[B11] Guo H. Chou W. C. Lai Y. Liang K. Tam J. W. Brickey W. J.. (2020). Multi-Omics Analyses of Radiation Survivors Identify Radioprotective Microbes and Metabolites. Science 370 (6516), 10. doi: 10.1126/science.aay9097 PMC7898465 33122357

[B12] Hall A. B. Yassour M. Sauk J. Garner A. Jiang X. Arthur T.. (2017). A Novel Ruminococcus Gnavus Clade Enriched in Inflammatory Bowel Disease Patients. Genome Med. 9 (1), 1–12. doi: 10.1186/s13073-017-0490-5 29183332 PMC5704459

[B13] Henke M. T. Kenny D. J. Cassilly C. D. Vlamakis H. Xavier R. J. Clardy J. (2019). Ruminococcus Gnavus, a Member of the Human Gut Microbiome Associated With Crohn’s Disease, Produces an Inflammatory Polysaccharide. Proc. Natl. Acad. Sci. 116 (26), 12672–12677. doi: 10.1073/pnas.1904099116 31182571 PMC6601261

[B14] Huang Z. Epperly M. Watkins S. C. Greenberger J. S. Kagan V. E. Bayır H. (2016). Necrostatin-1 Rescues Mice From Lethal Irradiation, (in Eng). Biochim. Biophys. Acta 1862 (4), 850–856. doi: 10.1016/j.bbadis.2016.01.014 26802452 PMC4788560

[B15] Jang H. Lee J. Park S. Kim J. S. Shim S. Lee S. B.. (2019). Baicalein Mitigates Radiation-Induced Enteritis by Improving Endothelial Dysfunction. Front. Pharmacol. 10, 892. doi: 10.3389/fphar.2019.00892 31474856 PMC6707809

[B16] Jianfeng G. Weiming Z. Ning L. Fangnan L. Li T. Nan L.. (2005). Serum Citrulline Is a Simple Quantitative Marker for Small Intestinal Enterocytes Mass and Absorption Function in Short Bowel Patients. J. Surg. Res. 127 (2), 177–182. doi: 10.1016/j.jss.2005.04.004 15921697

[B17] Lebeer S. Vanderleyden J. De Keersmaecker S. C. (2008). Genes and Molecules of Lactobacilli Supporting Probiotic Action. Microbiol. Mol. Biol. Rev. 72 (4), 728–764. doi: 10.1128/MMBR.00017-08 19052326 PMC2593565

[B18] Li K. Bihan M. Yooseph S. Methe B. A. (2012). Analyses of the Microbial Diversity Across the Human Microbiome. PLoS One 7 (6), e32118. doi: 10.1371/journal.pone.0032118 22719823 PMC3374608

[B19] Liang S. Deng X. Lei L. Zheng Y. Ai J. Chen L.. (2019). The Comparative Study of the Therapeutic Effects and Mechanisms of Baicalin, Baicalein and Their Combinations on Ulcerative Colitis Rat. Front. Pharmacol. 10, 1466. doi: 10.3389/fphar.2019.01466 PMC6923254 31920656

[B20] Liu Z.-Y. Wu B. Guo Y.-S. Zhou Y.-H. Fu Z.-G. Xu B.-Q.. (2015). Necrostatin-1 Reduces Intestinal Inflammation and Colitis-Associated Tumorigenesis in Mice. Am. J. Cancer Res. 5 (10), 3174. 26693068 PMC4656739

[B21] Macfarlane G. Gibson G. (1995). “Microbiological Aspects of Short Chain Fatty Acid Production in the Large Bowel”. in: Physiological and Clinical Aspects of Short Chain Fatty Acid Metabolism. Eds. Cummings J. H. Rombeau J. L. Sakata T. (Cambridge: Cambridge University Press), 87–105. ISBN 9780521440486

[B22] Macfarlane S. Macfarlane G. T. (2003). Regulation of Short-Chain Fatty Acid Production. Proc. Nutr. Soc. 62 (1), 67–72. doi: 10.1079/PNS2002207 12740060

[B23] Meshkibaf S. Martins A. J. Henry G. T. Kim S. O. (2016). Protective Role of G-CSF in Dextran Sulfate Sodium-Induced Acute Colitis Through Generating Gut-Homing Macrophages. Cytokine 78, 69–78. doi: 10.1016/j.cyto.2015.11.025 26687628

[B24] Osman M. A. Neoh H.-M. Ab Mutalib N.-S. Chin S.-F. Mazlan L. Ali R. A. R.. (2021). Parvimonas Micra, Peptostreptococcus Stomatis, Fusobacterium Nucleatum and Akkermansia Muciniphila as a Four-Bacteria Biomarker Panel of Colorectal Cancer. Sci. Rep. 11 (1), 1–12. doi: 10.1038/s41598-021-82465-0 33536501 PMC7859180

[B25] Pendyala S. Walker J. M. Holt P. R. (2012). A High-Fat Diet Is Associated With Endotoxemia That Originates From the Gut. Gastroenterology 142 (5), 1100–1101.e2. doi: 10.1053/j.gastro.2012.01.034 22326433 PMC3978718

[B26] Pols T. W. Noriega L. G. Nomura M. Auwerx J. Schoonjans K. (2011). The Bile Acid Membrane Receptor TGR5 as an Emerging Target in Metabolism and Inflammation. J. Hepatol. 54 (6), 1263–1272. doi: 10.1016/j.jhep.2010.12.004 21145931 PMC3650458

[B27] Quast C. Pruesse E. Yilmaz P. Gerken J. Schweer T. Yarza P.. (2013). The SILVA Ribosomal RNA Gene Database Project: Improved Data Processing and Web-Based Tools. Nucleic Acids Res. 41 (Database issue), D590–D596. doi: 10.1093/nar/gks1219 23193283 PMC3531112

[B28] Riva A. Kolimár D. Spittler A. Wisgrill L. Herbold C. W. Abrankó L.. (2020). Conversion of Rutin, A Prevalent Dietary Flavonol, by the Human Gut Microbiota. Front. Microbiol. 11, 3324. doi: 10.3389/fmicb.2020.585428 PMC7779528 33408702

[B29] Rwigema J.-C. M. Beck B. Wang W. Doemling A. Epperly M. W. Shields D.. (2011). Two Strategies for the Development of Mitochondrion-Targeted Small Molecule Radiation Damage Mitigators. Int. J. Radiat. Oncol. Biol. Phys. 80 (3), 860–868. doi: 10.1016/j.ijrobp.2011.01.059 21493014 PMC3104115

[B30] Sanna S. van Zuydam N. R. Mahajan A. Kurilshikov A. Vila A. V. Võsa U.. (2019). Causal Relationships Among the Gut Microbiome, Short-Chain Fatty Acids and Metabolic Diseases. Nat. Genet. 51 (4), 600–605. doi: 10.1038/s41588-019-0350-x 30778224 PMC6441384

[B31] Satyamitra M. Kumar V. P. Biswas S. Cary L. Dickson L. Venkataraman S.. (2017). Impact of Abbreviated Filgrastim Schedule on Survival and Hematopoietic Recovery After Irradiation in Four Mouse Strains With Different Radiosensitivity. Radiat. Res. 187 (6), 659–671. doi: 10.1667/RR14555.1 28362168 PMC5539877

[B32] Schaap F. G. Trauner M. Jansen P. L. (2014). Bile Acid Receptors as Targets for Drug Development. Nat. Rev. Gastroenterol. Hepatol. 11 (1), 55. doi: 10.1038/nrgastro.2013.151 23982684

[B33] Schloss P. D. Westcott S. L. Ryabin T. Hall J. R. Hartmann M. Hollister E. B.. (2009). Introducing Mothur: Open-Source, Platform-Independent, Community-Supported Software for Describing and Comparing Microbial Communities, (in Eng). Appl. Environ. Microbiol. 75 (23), 7537–7541. doi: 10.1128/AEM.01541-09 19801464 PMC2786419

[B34] Seregin S. S. Golovchenko N. Schaf B. Chen J. Pudlo N. A. Mitchell J.. (2017). NLRP6 Protects Il10–/– Mice From Colitis by Limiting Colonization of Akkermansia Muciniphila. Cell Rep. 19 (4), 733–745. doi: 10.1016/j.celrep.2017.03.080 28445725 PMC5528001

[B35] She Y.-Y. Kong X.-B. Ge Y.-P. Liu Z.-Y. Chen J.-Y. Jiang J.-W.. (2020). Periodontitis and Inflammatory Bowel Disease: A Meta-Analysis. BMC Oral. Health 20 (1), 1–11. doi: 10.1186/s12903-020-1053-5 PMC7069057 32164696

[B36] Sinha S. R. Haileselassie Y. Nguyen L. P. Tropini C. Wang M. Becker L. S.. (2020). Dysbiosis-Induced Secondary Bile Acid Deficiency Promotes Intestinal Inflammation. Cell Host Microbe 27 (4):659–670.e5. doi: 10.1016/j.chom.2020.01.021 PMC8172352 32101703

[B37] Stapleton A. L. Shaffer A. D. Morris A. Li K. Fitch A. Methé B. A. (2020). The Microbiome of Pediatric Patients With Chronic Rhinosinusitis. Int. Forum Allergy Rhinol. 11 (1), 31–39. doi: 10.1002/alr.22597 32348024

[B38] Steinman J. Epperly M. Hou W. Willis J. Wang H. Fisher R.. (2018). Improved Total-Body Irradiation Survival by Delivery of Two Radiation Mitigators That Target Distinct Cell Death Pathways. Radiat. Res. 189 (1), 68–83. doi: 10.1667/RR14787.1 29140165 PMC5808408

[B39] Tarabichi Y. Li K. Hu S. Nguyen C. Wang X. Elashoff D.. (2015). The Administration of Intranasal Live Attenuated Influenza Vaccine Induces Changes in the Nasal Microbiota and Nasal Epithelium Gene Expression Profiles. Microbiome 3 (1), 1–16. 26667497 10.1186/s40168-015-0133-2 PMC4678663

[B40] Thermozier S. Hou W. Zhang X. Shields D. Fisher R. Bayir H.. (2020). Anti-Ferroptosis Drug Enhances Total-Body Irradiation Mitigation by Drugs That Block Apoptosis and Necroptosis. Radiat. Res. 193 (5), 435–450. doi: 10.1667/RR15486.1 32134361 PMC7299160

[B41] Therneau T. M. Grambsch P. M. (2000). “The Cox Model,” in Modeling Survival Data: Extending the Cox Model (New York: Springer), 39–77. Available at: https://www.springer.com/gp/book/9780387987842.

[B42] Therneau T. M. Lumley T. (2014). Package ‘Survival’. Survival Anal. Published CRAN 2, 3.

[B43] Tiso M. Schechter A. N. (2015). Nitrate Reduction to Nitrite, Nitric Oxide and Ammonia by Gut Bacteria Under Physiological Conditions. PLoS One 10 (3), e0119712. doi: 10.1371/journal.pone.0119712 25803049 PMC4372352

[B44] Vítek L. Majer F. Muchová L. Zelenka J. Jirásková A. Branný P.. (2006). Identification of Bilirubin Reduction Products Formed by Clostridium Perfringens Isolated From Human Neonatal Fecal Flora. J. Chromatogr. B 833 (2), 149–157. doi: 10.1016/j.jchromb.2006.01.032 16504607

[B45] Wang W. Hu L. Chang S. Ma L. Li X. Yang Z.. (2020). Total Body Irradiation-Induced Colon Damage Is Prevented by Nitrate-Mediated Suppression of Oxidative Stress and Homeostasis of the Gut Microbiome. Nitric. Oxide 102, 1–11. doi: 10.1016/j.niox.2020.05.002 32470598

[B46] Wang Q. Garrity G. M. Tiedje J. M. Cole J. R. (2007). Naive Bayesian Classifier for Rapid Assignment of rRNA Sequences Into the New Bacterial Taxonomy. Appl. Environ. Microbiol. 73 (16), 5261–5267. doi: 10.1128/AEM.00062-07 17586664 PMC1950982

[B47] Xu M. Tao J. Yang Y. Tan S. Liu H. Jiang J.. (2020). Ferroptosis Involves in Intestinal Epithelial Cell Death in Ulcerative Colitis. Cell Death Dis. 11 (2), 1–13. doi: 10.1038/s41419-020-2299-1 32015337 PMC6997394

[B48] Zhang X. Fisher R. Hou W. Shields D. Epperly M. W. Wang H.. (2020). Second-Generation Probiotics Producing IL-22 Increase Survival of Mice After Total Body Irradiation. In Vivo 34 (1), 39–50. doi: 10.21873/invivo.11743 31882461 PMC6984118

