# Supplementary material for: “Longitudinal Fecal Microbiome Study of Total Body Irradiated Mice Treated With Radiation Mitigators Identifies Bacterial Associations With Survival”
Source: Front Cell Infect Microbiol. 2021 Sep 21;11:715396. doi: 10.3389/fcimb.2021.715396 (PMC8490782; doi:10.3389/fcimb.2021.715396)
Supplement: Supplementary file 1 [file Table_1.docx]

Supplementary Material

**1. Differences of microbiome composition based on paired-differences between time points.**

The values presented in Supplemental Tables 1, 2 and 3, were estimated with regression based on the paired-difference in the alr transformed abundances between time points from the same mouse. Supplemental Tables 1, 2 and 3, present the changes between “Baseline and Post-Irradiation” (Day 0 vs Day 1), “Post-Irradiation vs. Before First Sacrifice (Day 1 vs Day 7), and “Baseline and Survivors” (Day 0 vs Day 30), respectively. The results from the GSAA and IPCD Experiments are located on the left and right groups of columns, respectively. The groups of regression coefficients rows are located above the groups of p-values rows. **Coefficients with p-values < 0.01, are highlighted in red**, as an aid to help identify the most potentially interesting associations and to help identify potential trends across taxa or treatments. The treatments are represented across the columns and the taxa are represented across the columns. The “difference” column represents the control (intercept) of the regression model between the two time points of interest. A negative (or positive) “difference” represents a decrease (or increase) in the abundance from the earlier to later time point (respectively). The column values with the treatments indicate if there was an additional change between the time points, in addition to the “difference”. For example, in “Baseline vs Post-Irradiation”, the *Lactobacillus* cell in Experiment 1 the “difference” was -2.2064 and the p-value was 0.0000. This indicates that there was a drop in the abundance of *Lactobacillus* after irradiation. Since the treatment values were not statistically significant, it should be interpreted that the treatments were not significantly different from the control. The treatment groups had a similar drop in the magnitude and direction of *Lactobacillus* to the control, i.e. the treatments did not have an effect on the change of abundance between the time points. These matrices were provided to facilitate the examination of the relationships between specific treatments and taxa that is more difficult to grasp in prose.

The effect of irradiation on the mice prior to treatment is captured in Supplemental Table 1. The changes in the composition of the microbiota due to irradiation can be examined across the various treatment combination groups. Since the treatments were administered after the day 1 samples were taken, the tables document the variation that can be seen between experiments and also between groups of mice within the same experiment. Treatment group specific differences relative to the control are likely the result of cage effects. These tables also demonstrate the necessity of comparing time points to baseline to account for variations between batches of mice.

The change in the composition of the microbiota of the survivors (day 30) relative to their original baseline (day 0) composition is captured in Supplemental Table 3. In both experiments, there were only a few taxa (*Bacteroides* and *Bifidobacterium*) that were significantly different from baseline. Across the various treatments, there were few or no differences from the control. For example, the JP4-039+Baicalein treatment only had an additional increase in *Clostridium sensu stricto 1* more than the control.

Supplemental Tables 1-3 have also been provided in an Excel spreadsheet across multiple sheets.

**Supplemental Table 1. Paired-difference in taxonomic abundances between Baseline and Post-Irradiation.**

**Supplemental Table 2. Paired-difference in taxonomic abundances between Post-Irradiation and Before First Sacrifice (Day 1 vs Day 7).**

**Supplemental Table 3. Paired-difference in taxonomic abundances between Baseline and Day 30 of Survivors.**

**2. Cox proportional hazard regression results**

The Cox proportional hazards analysis produces an estimate for the hazard ratio for each of the variables (taxa and treatments) included in the survival model. A hazard ratio (HR), exp(coef), greater than 1 implies that the influence of the variable is deleterious. A hazard ratio less than 1, implies that the influence of the variable improves the survival of the subject. The following table (Supplemental Table 3) allows the reader to compare the results between the two experiments 1 and 2 (left and right column groups) and between the Full and Reduced models (upper and lower row groups). A comparison between the two experiments allows the reader to examine where the top 15 fecal taxa were different and how consistent the estimated hazard ratios were. Contradictory estimated HRs can be examined for their statistical significance. Similarly, when statistical significance is marginal but in agreement between experiments, further inquiry may be justified. The comparison between the Full and Reduced model allows the reader to examine how the HR changes for variables in the Reduced model (treatments only), when components of the microbiome are included into the Full model (treatment + microbiome). If the model had not been overfit, i.e. including more variables than can be supported by the sample size, and the addition of the microbiota variables reduces the significance of the treatment’s HR, then the microbiota augments the model’s explanatory power of survival relative to the treatment alone. Based on the results below, the significance of the HR were not completely eliminated, suggesting that both the treatments and microbiome had a cumulative impact on the subject’s survival. This implies there is value in examining the contribution of the microbiome towards understanding the mice’s survival.

Supplemental Table 4 has also been provided in an Excel spreadsheet.

#

**Supplemental Table 4. Cox proportional hazards regression coefficients comparing experiments and models.** The Cox proportional hazards model is a linear model when the hazard ratio is logarithmically transformed. Thus, estimated coefficients (coef) must be exponentiated (exp) to reproduced the hazard ratio (exp(coef)). The standard error (se(coef)), z statistic (z), and p-values (Pr(>|z|)) are estimated on the coefficients (before exponentiation), but the 95% confidence intervals (LB 95% = lower bound, UB 95% = upper bound), are provided as hazard ratios.
